# 3D printing guide plate for accurate hemicortical bone tumor resection in metaphysis of distal femoral: a technical note

**DOI:** 10.1186/s13018-021-02374-w

**Published:** 2021-05-28

**Authors:** Hongwei Wu, Shuo Yang, Jianfan Liu, Linqin Li, Yi Luo, Zixun Dai, Xin Wang, Xinyu Yao, Feng Zhou, Xian’an Li

**Affiliations:** Department of Orthopedics, Hunan Cancer Hospital and The Affiliated Cancer Hospital of Xiangya School of Medicine, Central South University, 283 Tongzipo Road, Changsha, Hunan 410013 People’s Republic of China

**Keywords:** Bone resection, Metaphysis of the distal femur, Reconstruction, Devitalization, 3D guide plate, Hemicortical resection

## Abstract

**Background:**

Surgical resection and reconstruction for low-grade bone sarcoma in the metaphysis of the distal femur remain challenging. We hypothesized that 3D printing osteotomy guide plate could assist to accurately resect the tumor lesion and save the joint function.

**Methods:**

From January 2017 to August 2019, five patients diagnosed with low-grade bone sarcoma in the metaphysis of the distal femur were treated with hemicortical resection using 3D printing guide plate. Autologous bone graft was inactivated in a high-temperature water bath and re-implanted in situ fixed with plate and screw. Patients were followed up from 17 to 33 months. The Musculoskeletal Tumor Society Score was used to evaluate the joint function. X-ray was used to evaluate the bone union.

**Results:**

One patient was paracorticular osteosarcoma, and four cases had highly differentiated chondrosarcoma. All cases were involved in the metaphysis of the distal femur. Patients were followed up from 13 to 33 months, with an average of 23.6 months. There was neither post-operation infection, internal fixation loosening, nor fracture occurrence in any of the patients. The Musculoskeletal Tumor Society Score averaged at 28.1, while the International Society of Limb Salvage imaging score examination averaged 89.8%.

**Conclusions:**

Here, we demonstrate that the 3D printing osteotomy guide plate-assisted hemicortical bone resection is a beneficial strategy to effectively resect the primary low-grade malignant bone tumors in the metaphysis of the distal femur and retained satisfied joint function.

**Supplementary Information:**

The online version contains supplementary material available at 10.1186/s13018-021-02374-w.

## Background

Paracortical osteosarcoma and highly differentiated chondrosarcoma are the most prevalent low-grade malignant primary bone tumors [1, 2]. Hemiexcision of the tumor bone with inactivated tumor bone replantation is a valuable surgical procedure for low-grade malignant bone tumors [3–5]. However, it has been reported that this procedure has several complications, such as inadequate margins, infection, and fractures of the host bone [6]. Especially in the metaphysis of the long bone, unintended excision margin usually resulted in dysfunction of the adjacent joint [5]. For safe surgical boundaries, the hemibone resection strategy should retain as more normal bone tissue as possible, thus providing favorable conditions for the reconstruction of the bone defects for the rapid postoperative recovery [6–8].

Recently, 3D printing technology has been extensively used in the medical field [9–12]. For instance, establishing tumor models in vitro helps doctors understand tumor shapes and the adjacent tissues from multiple perspectives and also identify lesion locations and surgical risks. Additionally, the technology has been applied in 3D printing implants through in vivo studies to assist in determining surgical boundaries [13–15]. In this technical note, we describe the technique using 3D printed osteotomy guide plate for accurate hemiexcision. Short-term follow-up results and shortcomings of this technique were also discussed.

## Methods

Five patients with tumor mass in the distal metaphysis of the femur were selected to receive this operation from January 2017 to August 2019 (Table 1). There were four cases of posterior femoral tumor and one case of lateral femoral tumor. One patient was diagnosed with paracortical osteosarcoma, and the other four were diagnosed with chondrosarcoma. The diagnosis was taken via biopsy and reported by a specialized pathologist. There were one male and four females. The minimum age of the patients was 11, the maximum 65, and the mean age was 29.8 years old.

**Table 1:** Clinical characteristics of patients who received hemicortical resection and reconstruction

No	Age/gender	Diagnosis	Site	Resected bone (cm)	% of cortical circumference	Fixation
1	11/F	PO	Femur	11	40	P+S
2	37/M	CS	Femur	9	40	P+S
3	17/F	CS	Femur	5	30	P+S
4	65/F	CS	Femur	15	50	P+S
5	19/F	CS	Femur	13	30	P+S

*PO* paracortical osteosarcoma, *CS* chondrosarcoma, *P+S* plate and screw

**Fig. 1:**
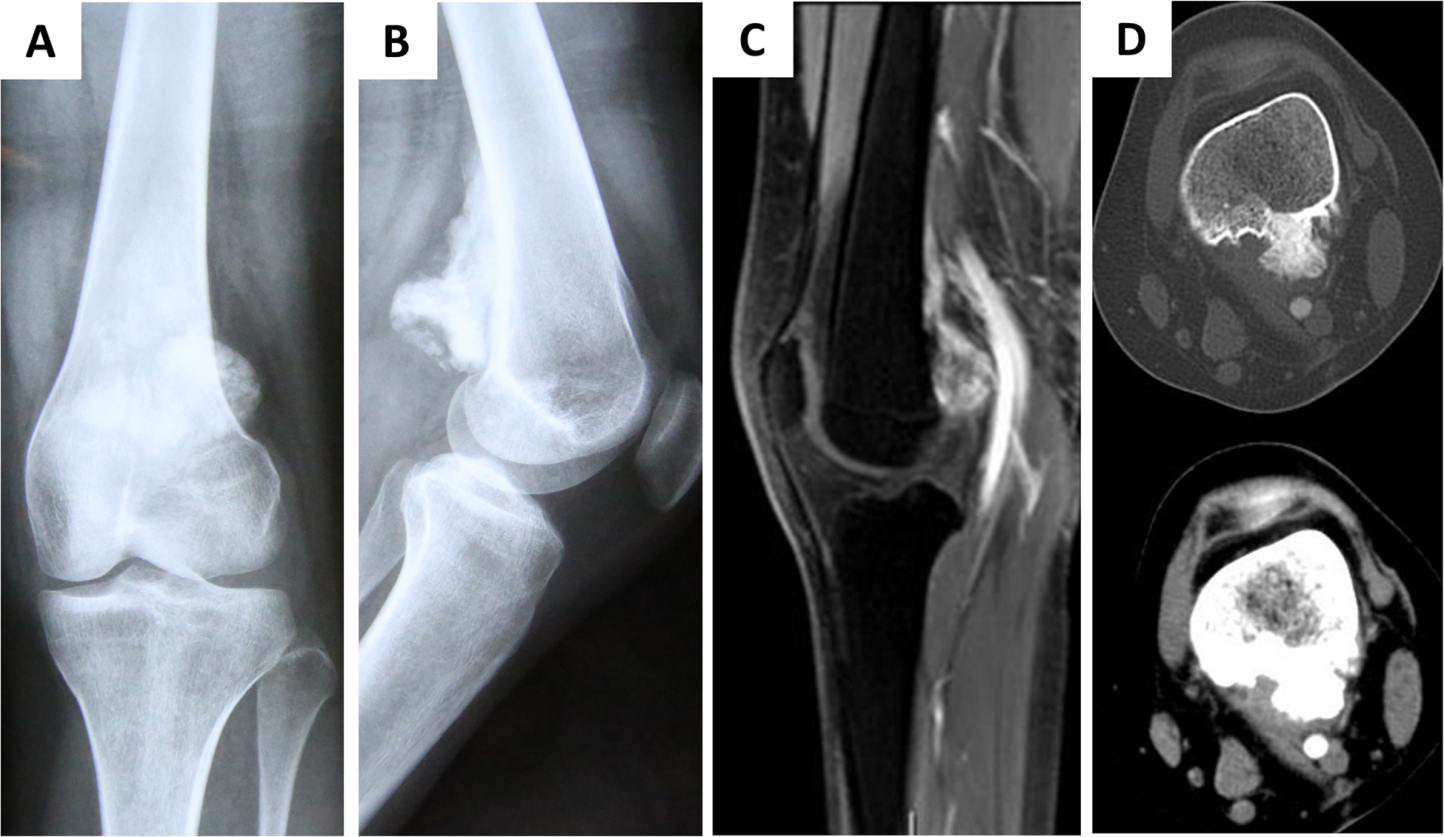
Image features of chondrosarcoma in the metaphysis of the distal femur. Nineteen-year-old female patient with chondrosarcoma. **a**, **b** X-ray indicated a large osteogenic bone lesion at the posterior femur of the left leg. **c** MRI enhanced scan showing the lesion was closely adjacent to the popliteal vessels. **d** Cross-section of CT scan demonstrated that the lesion was close next to the vessels

X-ray, thin-layer enhanced three-dimensional CT, and MRI were conducted for the tumor site examination upon admission (Fig. 1). The patients were informed of the feasibility of this operation based on the comprehensive assessment of their medical history, clinical signs, and imaging manifestations.

**Fig. 2:**
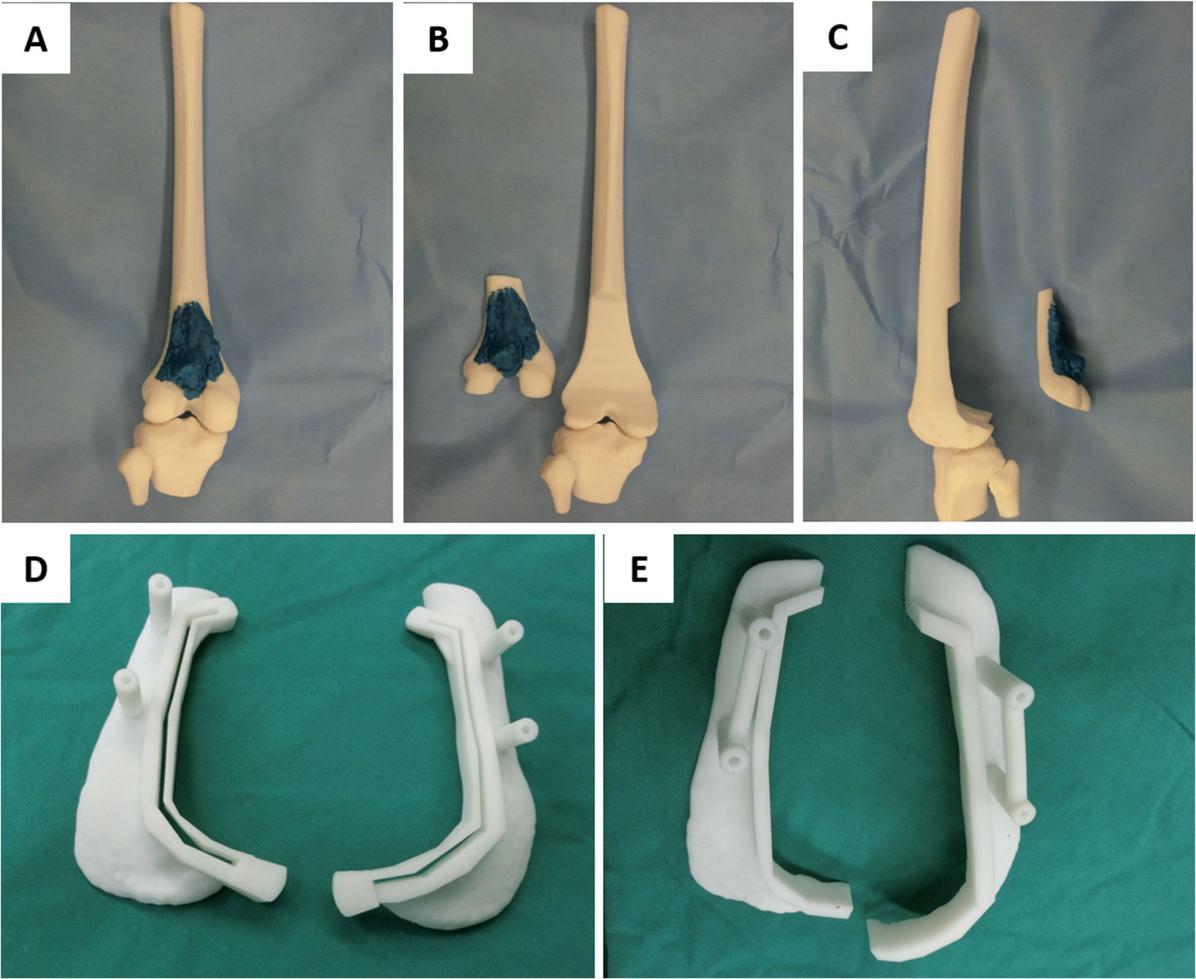
3D printed bone tumor model and guide plate. **a** 3D printed bone tumor model. **b**, **c** The planned excision boundary of the lesion. **d**, **e** 3D printed bone resection guide plate with guide pin holes

Simultaneously, we retrieved the enhanced CT scan data of the bone tumor sites and used the Mimics software (Materialise’s interactive medical image control system, USA) to extract and reconstruct the 3D CT scan data, to highlight the location and size of the tumor lesions, and to print the 3D tumor bone model. We used the Mimics software to mark the tumor resection scope and the osteotomy model (Fig. 2a–c). Afterward, the doctor determined the safe boundary of tumor resection. For low-grade malignant tumors, 2 cm outside the tumor was generally selected as the safe boundary of resection. The osteotomy guide plate was then developed to facilitate the determination of the resection boundary and efficacy of accurate osteotomy during the operation (Fig. 2d, e). The tumor bone model and the guide plate were both printed with poly-l-lactide acid (PLLA) using the melting extrusion method.

**Fig. 3:**
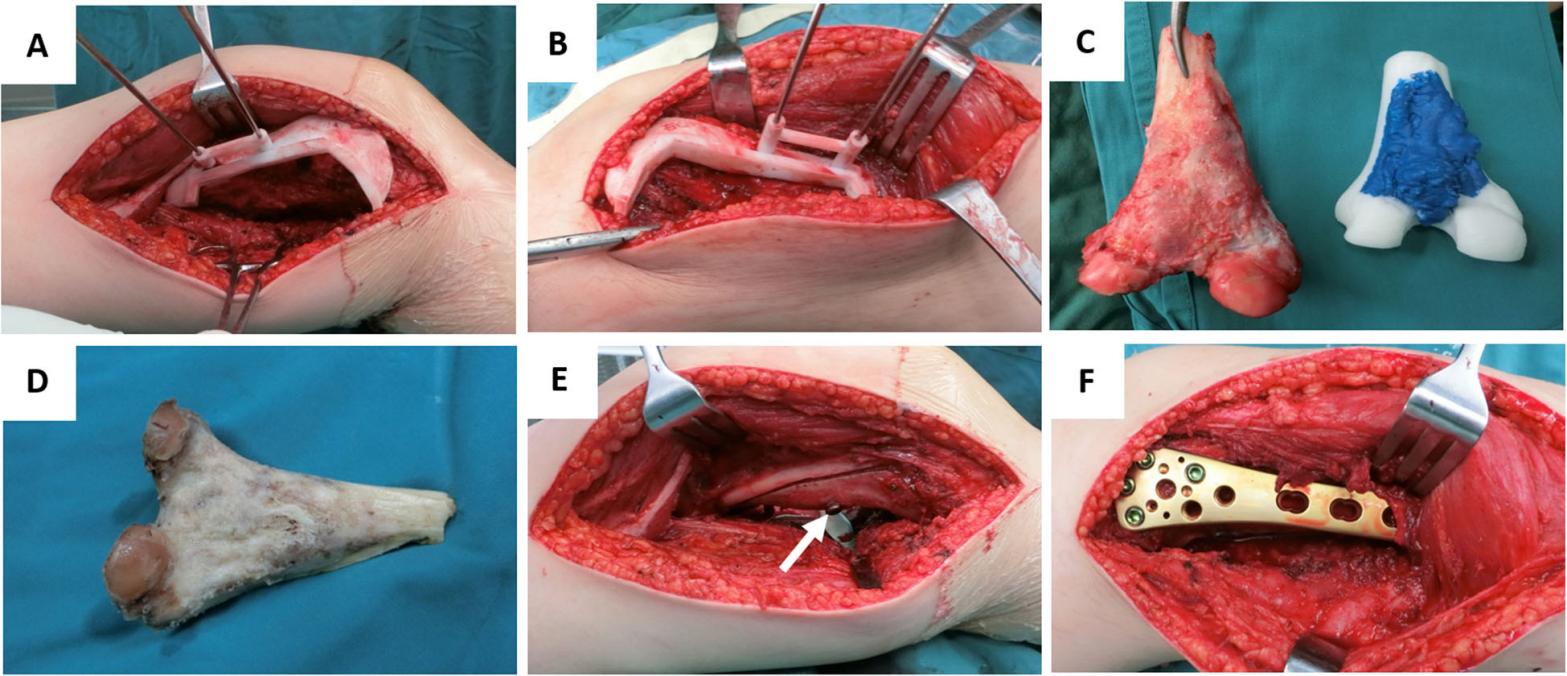
Operation procedure for the resection of the tumor. **a** 3D printing guide plate fixed by two K-wires in the medial side. **b** 3D printing guide plate fixed by two K-wires on the lateral side. **c**, **d** The tumor block was resected intactly and deactivated. **e** The deactivated bone graft was re-implanted orthotopically. **f** The bone graft was fixed by screw and plate

During the operation, we adopted general anesthesia and optimal surgical approaches based on the different tumor sites. For the posterior femoral tumor, dual medial and lateral incisions were adopted to completely expose the operative field [4]. The surface of the mass was generally covered with a fibrous capsule. The capsule was not cut directly, while the normal tissue outside the capsule was separated to the cortical surface of the bone. The vessels close to the tumor were separated apart from the mass and protected carefully. The periosteum stripper stripped the adjacent soft tissue attached to the bone about 3–5 cm up and down. The osteotomy guide plate was then covered on the surface of the tumor bone and fixed by two K-wires (Fig. 3a, b), and the pendulum sawing osteotomy along the guiding plate was applied to remove the whole tumor bone. During the process of osteotomy, the saw should be maintained accurately along the groove to prevent the breakage of the guide plate. We scraped the visible tumor bone tissue using the curettage and retained the cutting edge for routine disease examination. We treated the remaining bone tissue using normal saline at 70 °C for 30 min to inactivate the tumor cells in the bone tissue (Fig. 3c, d). Subsequently, the inactivated bone tissue was transplanted back to the bone defect site, and the appropriate length of the screw and bone plate was selected to fix the inactivated bone and the adjacent normal bone tissue (Fig. 3e, f).

At 3, 6, 12, and 24 months after the operation, we performed regular follow-up for X-ray to check the bone graft situation and the abdominal B-ultrasound and chest CT to assess whether there was distant metastasis. We adopted the American Society for bone tumors (Musculoskeletal Tumor Society (MSTS)) function scoring system to score [16] the limb function after the reconstructive surgery. We also used the international limb-salvage association (International Society of Limb Salvage (ISOLS)) imaging scoring system for the evaluation of the patients with postoperative radiographic images regarding the bone healing at the cutting surface, the graft bone changes, the stability of the internal fixation, and the joint mobility. Lastly, the percentage evaluation result was obtained by dividing the sum of the integral of each item by the full score.

## Results

All patients received a complete resection of the bone tumor. In particular, the longest inactivated bone tumor after resection was 17 cm, while the shortest bone tumor was 9 cm, with an average of 13.8 cm. The excised bone accounted for 30–50% of the diameter of the diaphysis, with an average of 38%. Notably, postoperative pathological analysis was consistent with the biopsy pathology results.

**Fig. 4:**
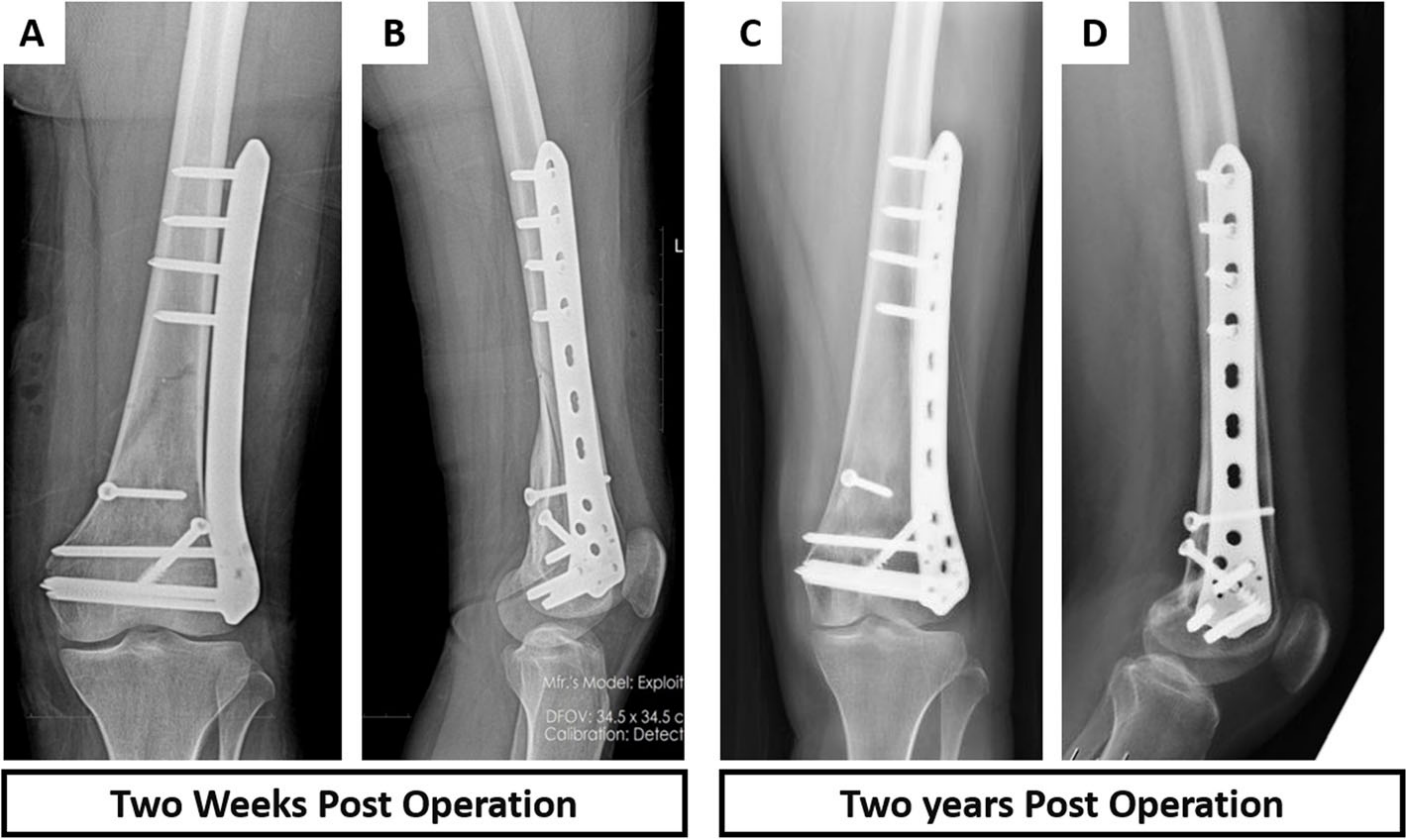
Follow-up images of the representative patient. **a**, **b** X-ray images 2 weeks post-operation showing that the gap was clear between the bone block and the host bone. **c**, **d** X-ray images 24 months post-operation

Particularly, there were four cases with a giant lesion very close to the posterior tibial vessels and femur condyles (see supplemental materials). Besides the vessels were delicately separated from the lesion, the lesion was resected intactly using this method (Fig. 3c, d). The inactivated bone was then re-implanted into the defect site. The fixation was stable, and the bone graft was well fixed (Fig. 3e, f). Additionally, no residual tumor at the postoperative incision edge was noted in any patient based on the pathological examination. Overall, the pain score at 3 months after surgery was 1–4 points, averaging 2.2 points. All patients were followed up after surgery for 17–33 months, and the median follow-up time was 23.6 months. The highest percentage of the resected bone was 50% of the cortical circumference, and the lowest one was 30%. Importantly, none of the patients developed loosening or fracture of the internal fixation and femur fracture from the post-operation X-ray examination. The case in this technical note exhibited bone union 24 months post-operation (Fig. 4c, d).

Patients were suggested to walk cautiously with the aid of crutches 3 months after surgery. During the last follow-up, all patients exhibited satisfactory limb function and good joint activity. Representative images and videos were shown in supplementary materials. The MSTS score averaged at 28.1, while the ISOLS imaging score examination averaged 89.8%. Of note, there was no postoperative recurrence, internal fixation loosening and fracture, bone mass displacement, and metastasis in all patients (Table 2).

**Table 2:** Clinical outcome of five patients with hemicortical resection and reconstruction

No.	Age/gender	Complications/fixation failure	Pain score	FWB (months)	Follow-up (months)	MSTS Score	ISOLS Score	Recurrence/metastasis
1	11/F	Incision exudation/N	4	3	18	27	83	N/N
2	37/M	Wound effusion/N	4	3	17	26	78	N/N
3	17/F	None/N	1	3	33	27	91	N/N
4	65/F	None/N	2	3	26	29	93	N/N
5	19/F	Wound effusion/N	1	3	24	29	94	N/N

*FWB* full weight-bearing, *MSTS* Musculoskeletal Tumor Society, *ISOLS* International Society of Limb Salvage, *N* negative


Case 5.


Case 5.

## Discussion

In this technical note, we introduced an effective method to resect the low-grade bone tumor in the metaphysis of distal femur intactly. Conventional hemicortical excision or piece meal resection usually resulted in residual tumor or fracture. The resection plane could not be controlled delicately. Especially in the posterior part of the distal femur, the vessels usually interfere with the exposure of the surgical field, making it more difficult to operate. Furthermore, excision of large segments of the joint requires extensive joint reconstruction. Allogenic bone graft or cement for this reconstruction leads to reduced joint function and a similar risk of infection to other internal plants [17, 18]. Therefore, despite its limited indications, hemicortical resection followed by inactivation and replantation is recognized and applied by many scholars [6–8, 19]. Campanacci et al. were the first to report the application of hemiectomy in bone tumor surgery [20]. Also, some scholars have used hemiexcision for the surgical treatment of high-grade osteosarcoma. It has been noted that it is suitable for eccentric bone tumors, but its long-term effects remain elusive [19]. In particular, hemiexcision is more predominantly used for low-grade malignant tumors [3, 4, 7, 21, 22]. In this study, all the patients were of low-grade malignant tumors. The benefits of hemiexcision include tumor resection can be expanded, preserves the stability and integrity of adjacent joints, enhances the residual normal bone mechanics using autologous or allogeneic bone grafting, matches the size of the original bone defect accurately, and has no risk of disease transmission. Of note, the initial safety margin of tumor resection is crucial to the treatment effect. Factors such as tumor location, shape, and size pose challenges to the effective application of hemibone resection. Due to the irregular shape of the tumor, and the restriction of the surgical field of view, surgeons may have to make certain plan changes during the osteotomy procedure. This may lead to the unsafe tumor resection border and the recurrence of residual tumor. For malignant bone tumors at the distal end of the posterior femur, it is difficult to preserve the blood vessels as well as joint function [4].

In the past, no method was available to make three-dimensional measurements on the tumor before surgical resection. However, computer technology has enabled this measurement to be made, thus achieving a higher matching degree between the tumor defect removed and the bone graft reconstructed [23, 24]. Another method used to accurately remove a tumor from the bone is the surgical navigation robot, but this tool is expensive and not readily available in ordinary hospitals. Recently, 3D digital reconstruction and 3D printing of osteotomy guide plate technology have improved osteotomy for hemibonectomy of bone tumors. The 3D reconstruction of the bone tumor is achieved using a three-dimensional CT scan which transmits the data into a 3D reconstruction software to establish a 3-dimensional model. This technique reveals the tumor after printing, thus allowing doctors to make a plan for the resection border. The corresponding resection guide plate can be fabricated according to the plan [23]. Theoretically, the safe boundary of osteotomy for this method is more reliable, and the chances of postoperative recurrence are lower. Moreover, with the assistance of the osteotomy guide plate, the time needed for osteotomy localization is relatively shorter, which reduces the operation time and in turn decreases blood loss, thus making it more effective in the rehabilitation of patients. Using the osteotomy guide plate, most of the bone tumor can be removed as a whole, rather than unplanned lumps. After inactivation treatment, the original shape of most of the bone tumor is maintained which creates a very high matching degree with the bone defect site. This ensures good fixation of bone blocks and also shortens the operation time. Overall, this method results in good postoperative recovery and functional recovery.

Previous studies have reported that fractures, infections, and incomplete resection contribute to the development complications of hemiexcision. Specifically, fracture is one of the leading cause (10–18%) [3, 25]. In 2014, some scholars used computer-assisted surgery to design an allograft bone graft to repair the bone defect of hemibonectomy. They noted that the method achieved resection and reconstruction precisely with less time-consuming and also reduced the incidence of fracture [23]. Therefore, a 3D-printed osteotomy guide plate can be used to perform accurate osteotomy based on the preoperative surgical plan, without any intraoperative or postoperative fractures. Herein, the inactivated bone tissue was transplanted back into the patient perfectly matched with the original bone defect, shortening the time taken to reconstruct the bone defect and adjust the bone mass. Besides, our short-term postoperative follow-up results enumerated no recurrence in all 5 patients. This implies a safe tumor resection boundary. We also achieved accurate R0 resection and successful reconstruction even for adjacent joint lesions, and this perhaps may have contributed to the highly preserved joint function. Functional scores of the affected limbs after surgery were above 24 points in all patients, while patient satisfaction was very high. This finding is consistent with the recent reports by Japanese scholars [26] and also exceeds scores reported in a review by Dutch scholars [6]. Therefore, this surgical approach is effective for the removal of bone tumors and bone reconstruction. High short-term efficacy following inactivation and replantation of hemibone resection for highly malignant bone tumors has been reported [19]. This indicates that it is possible to achieve an effective safe boundary for tumor control. However, the long-term efficacy of our method should be investigated further to confirm these intriguing findings, especially in follow-up studies.

In summary, digital three-dimensional reconstruction is a valuable technique for formulating osteotomy boundary and osteotomy guide plate-assisted osteotomy, which makes hemibone resection more convenient, faster, and reduces the risk of postoperative complications, and lowers the recurrence rate. Furthermore, the method used herein is cheap, reliable, and results in quick recovery. Notably, this is important for reserving the joint function regarding the tumor adjacent to the joint. Despite these benefits, we acknowledge that hemiexcision has its inherent limitations. Among them is the risk of postoperative recurrence and insecure surgical boundaries. The follow-up period for patients in this study is relatively short, and thus, a longer follow-up duration is needed to test the long-term effect of this surgical method. Also, the current digital three-dimensional reconstruction does not accurately identify the tumor tissue, making it difficult to achieve intelligent grasp recognition. Therefore, manual intervention is required to determine the tumor boundary. We believe that with further advancements in imaging, digital technology, and artificial intelligence, these problems will be gradually solved, and hemibonectomy will yield better therapeutic effects.

## Conclusions

The 3D printing guiding plate offered a useful approach for completely resecting low malignant metaphyseal bone tumors. The joint function is excellent, and the recurrence rate is low for short-term follow-up. The technique saves time and decreases bleeding during the operation. The cost of this technic is affordable and worthy to be applied to this kind of disease.

## Supplementary Information

**Additional file 1.** Case 1. Case 2. Case 3. Case 4. Case 5 po 3 months.

**Additional file 2.** Supplement figure.

## Data Availability

All the data and materials are available from the corresponding author upon reasonable request.

## Abbreviations

FWB: Full weight-bearing
MSTS: Musculoskeletal Tumor Society
ISOLS: International Society of Limb Salvage
N: Negative
PO: Paracortical osteosarcoma
CS: Chondrosarcoma
P+S: Plate and screw
