# Supplementary figures and images for: 3D printing guide plate for accurate hemicortical bone tumor resection in metaphysis of distal femoral: a technical note
Source: J Orthop Surg Res. 2021 May 28;16:343. doi: 10.1186/s13018-021-02374-w (PMC8161929; doi:10.1186/s13018-021-02374-w)

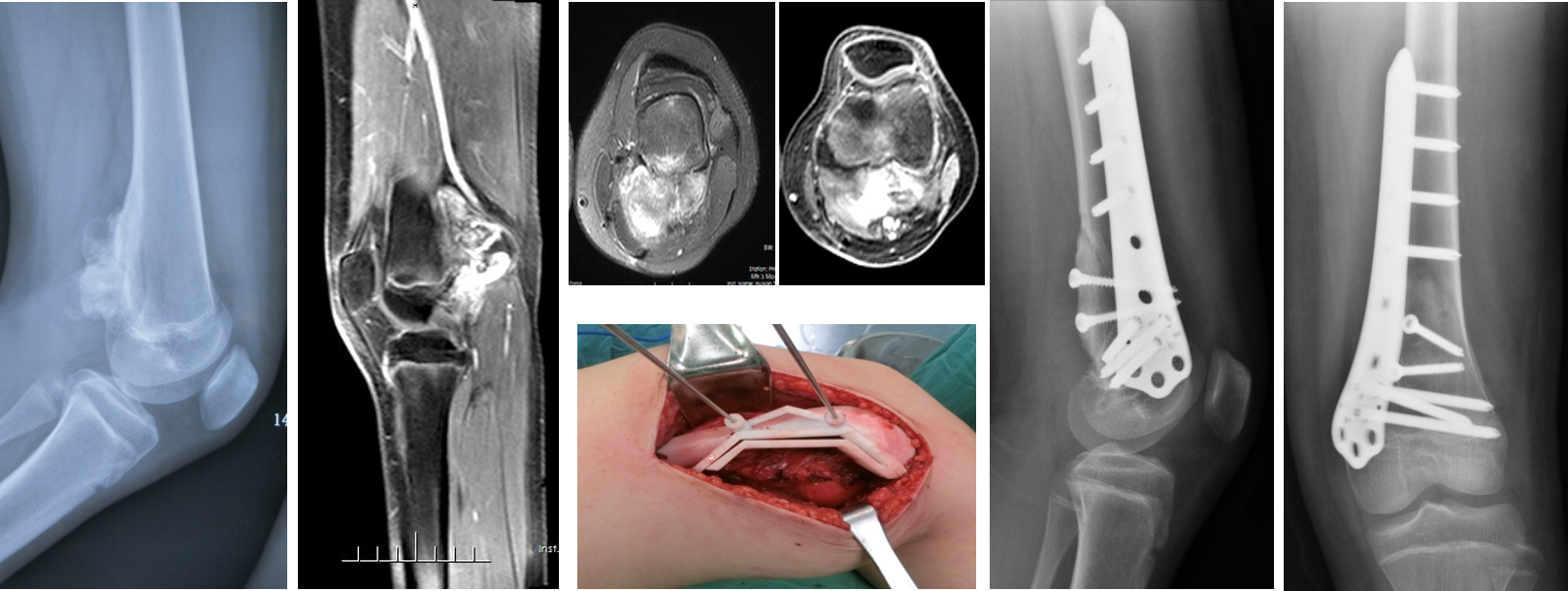

Supplement: Supplementary file 3 — Additional file 1. Case 1. Case 2. Case 3. Case 4. Case 5 po 3 months. [file 13018_2021_2374_MOESM1_ESM.zip › case 1 Li.png]

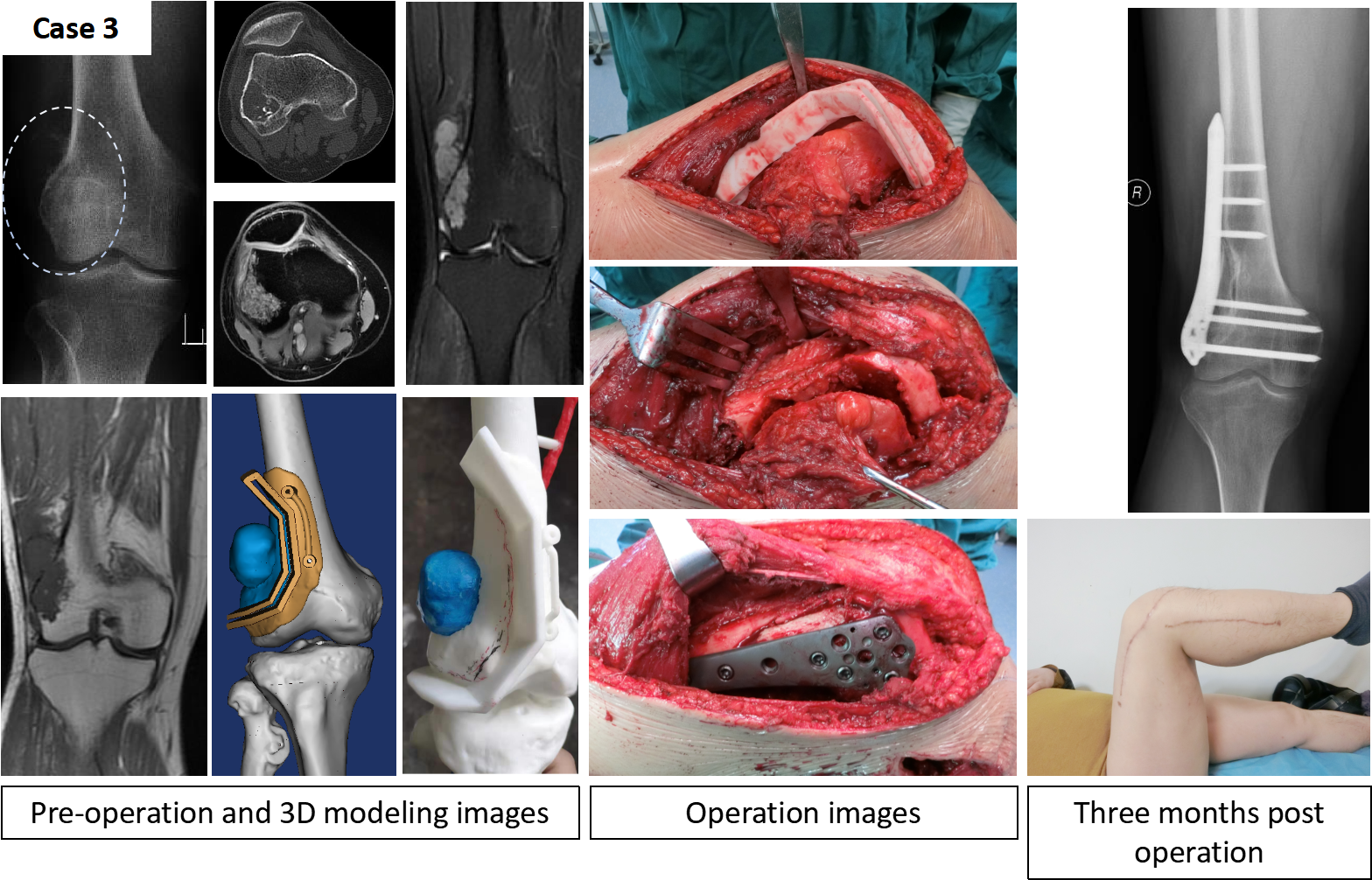

Supplement: Supplementary file 3 — Additional file 1. Case 1. Case 2. Case 3. Case 4. Case 5 po 3 months. [file 13018_2021_2374_MOESM1_ESM.zip › Case 3 Li.png]

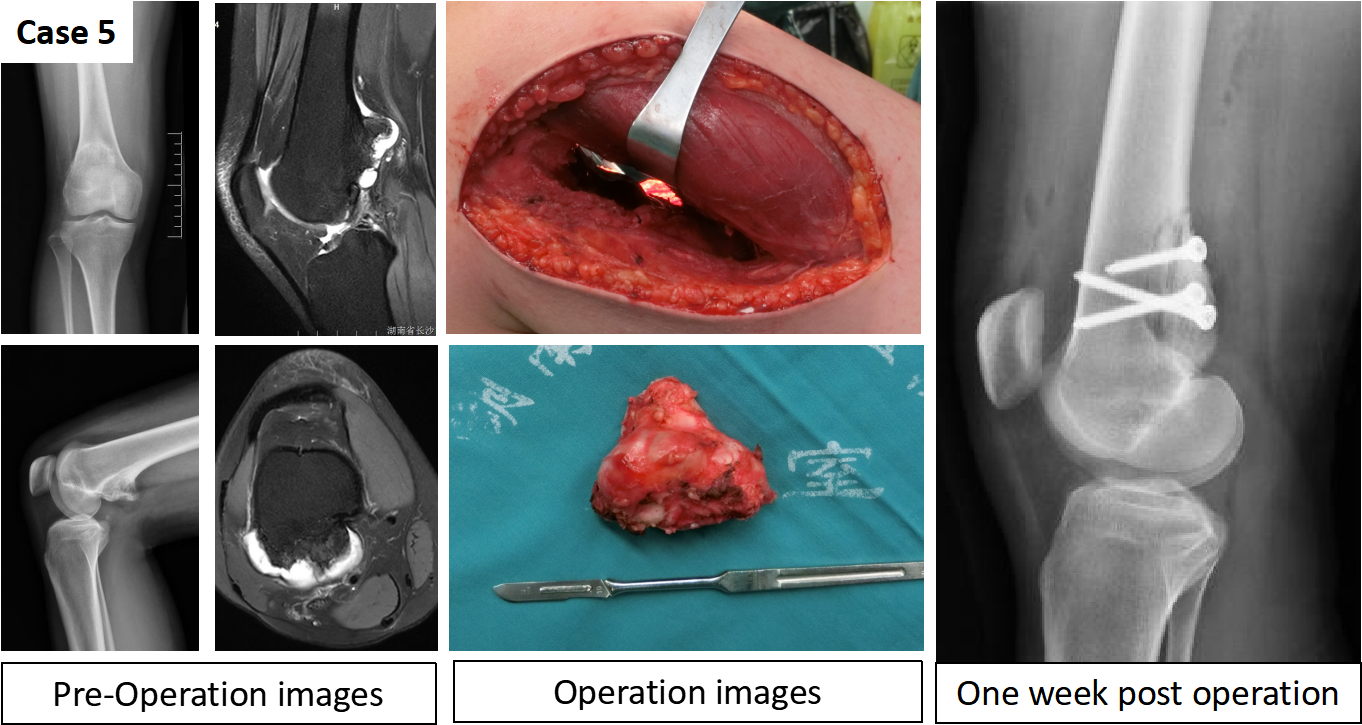

Supplement: Supplementary file 3 — Additional file 1. Case 1. Case 2. Case 3. Case 4. Case 5 po 3 months. [file 13018_2021_2374_MOESM1_ESM.zip › case 5 You.png]

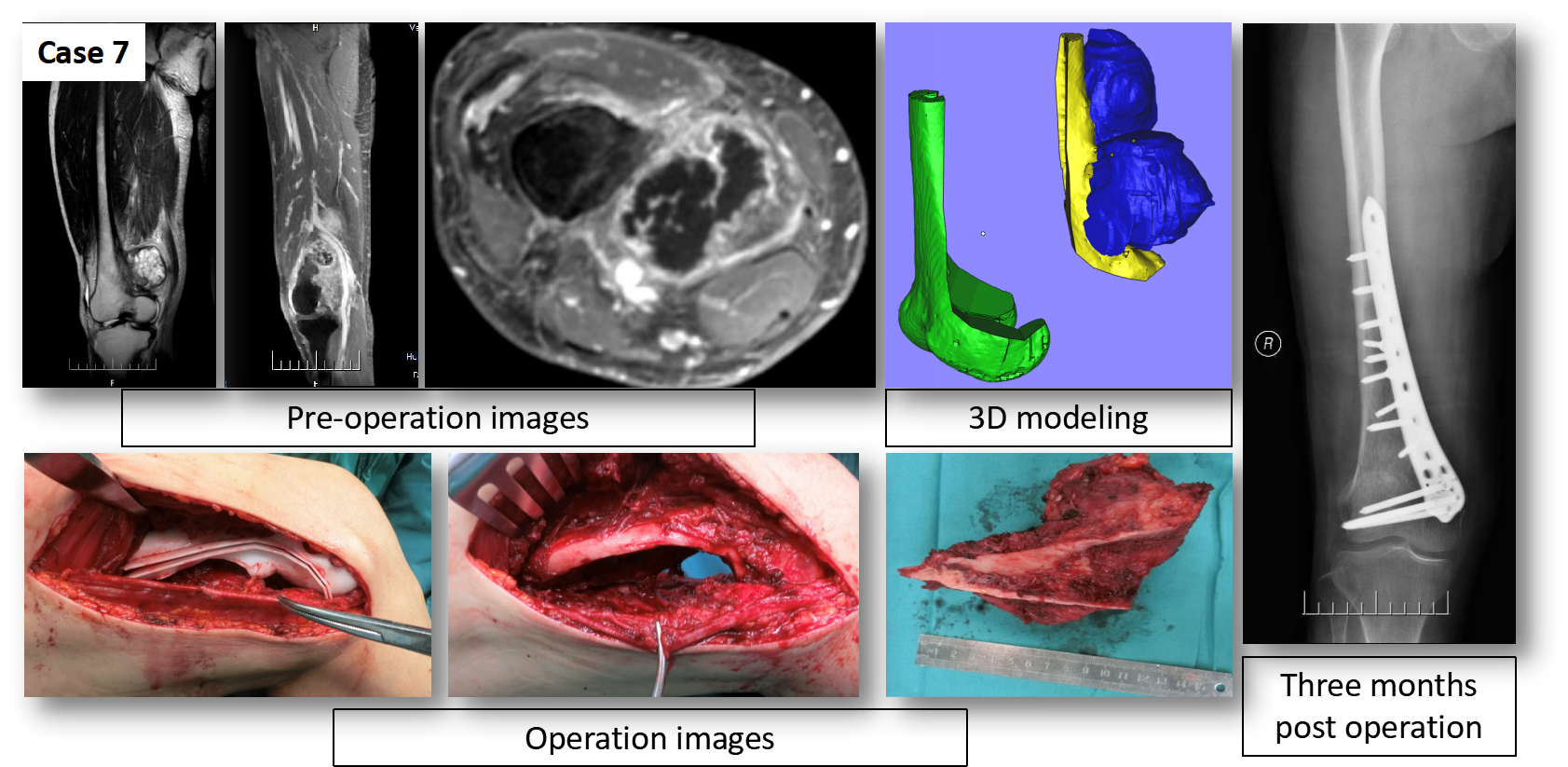

Supplement: Supplementary file 3 — Additional file 1. Case 1. Case 2. Case 3. Case 4. Case 5 po 3 months. [file 13018_2021_2374_MOESM1_ESM.zip › case 7 Xiao.png]

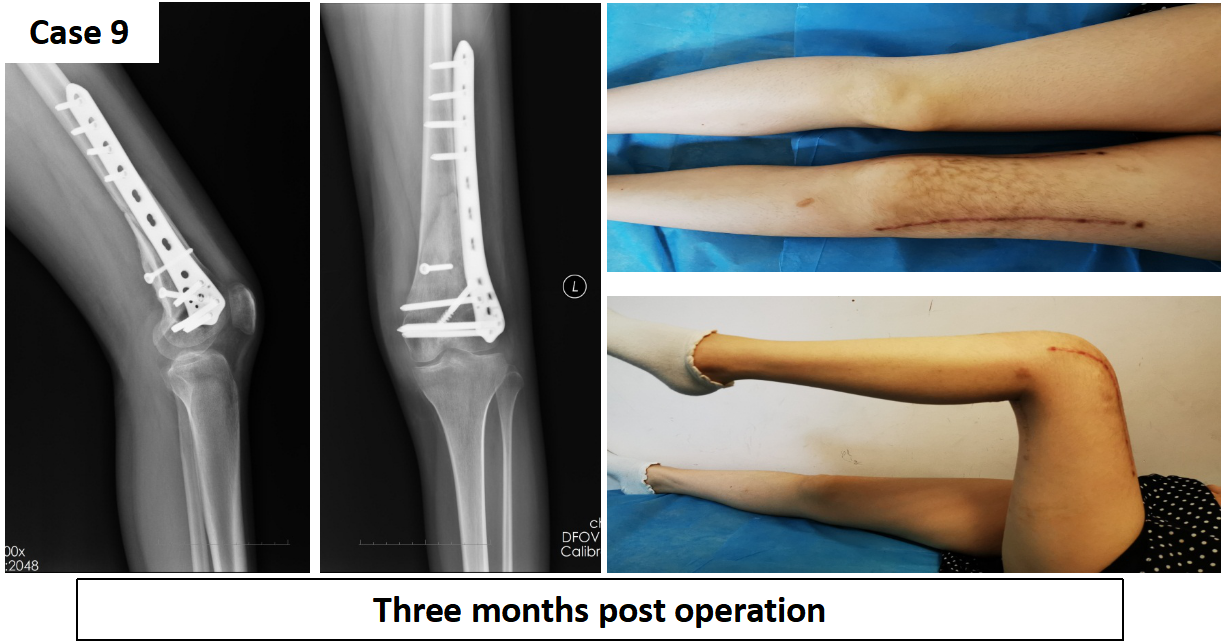

Supplement: Supplementary file 3 — Additional file 1. Case 1. Case 2. Case 3. Case 4. Case 5 po 3 months. [file 13018_2021_2374_MOESM1_ESM.zip › case9 po 3 months.png]

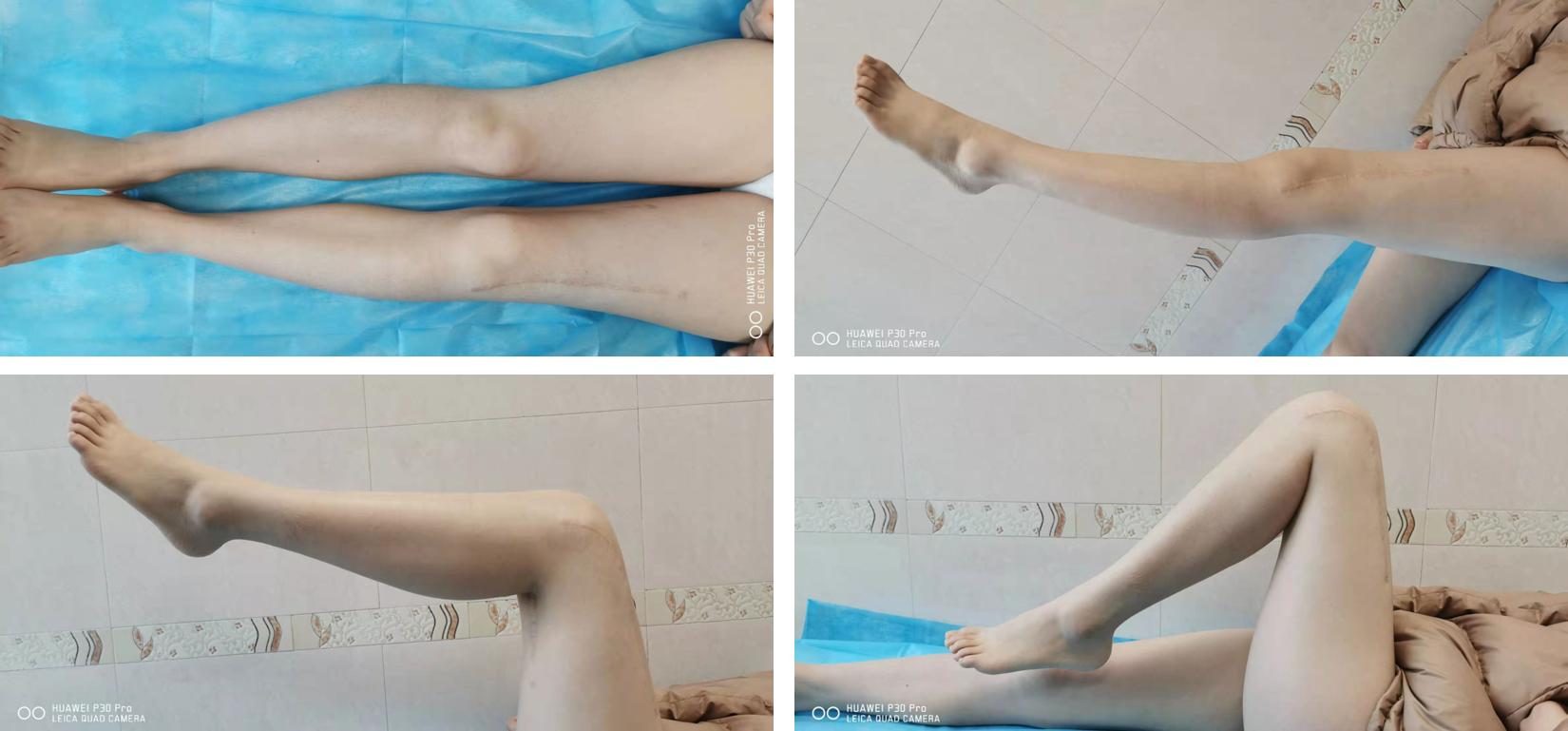

Supplement: Supplementary file 4 — Additional file 2. Supplement figure. [file 13018_2021_2374_MOESM2_ESM.png]
